# Dehydroamino acid residues in bioactive natural products

**DOI:** 10.1039/d3np00041a

**Published:** 2023-11-09

**Authors:** Shan Wang, Kewen Wu, Ya-Jie Tang, Hai Deng

**Affiliations:** a State Key Laboratory of Microbial Technology, Shandong University Qingdao 266237 China shan.wang@sdu.edu.cn yajietang@sdu.edu.cn; b Department of Chemistry, University of Aberdeen Aberdeen AB24 3UE UK h.deng@abdn.ac.uk

## Abstract

Covering: 2000 to up to 2023

α,β-Dehydroamino acids (dhAAs) are unsaturated nonproteinogenic amino acids found in a wide array of naturally occurring peptidyl metabolites, predominantly those from bacteria. Other organisms, such as fungi, higher plants and marine invertebrates, have also been found to produce dhAA-containing peptides. The α,β-unsaturation in dhAAs has profound effects on the properties of these molecules. They display significant synthetic flexibility, readily undergoing reactions such as Michael additions, transition-metal-catalysed cross-couplings, and cycloadditions. These residues in peptides/proteins also exhibit great potential in bioorthogonal applications using click chemistry. Peptides containing contiguous dhAA residues have been extensively investigated in the field of foldamers, self-assembling supermolecules that mimic biomacromolecules such as proteins to fold into well-defined conformations. dhAA residues in these peptidyl materials tend to form a 2.0_5_-helix. As a result, stretches of dhAA residues arrange in an extended conformation. In particular, peptidyl foldamers containing β-enamino acid units display interesting conformational, electronic, and supramolecular aggregation properties that can be modulated by light-dependent *E*–*Z* isomerization. Among approximately 40 dhAAs found in the natural product inventory, dehydroalanine (Dha) and dehydrobutyrine (Dhb) are the most abundant. Dha is the simplest dehydro-α-amino acid, or α-dhAA, without any geometrical isomers, while its re-arranged isomer, 3-aminoacrylic acid (Aaa or ΔβAla), is the simplest dehydro-β-amino acid, or β-enamino acid, and displays *E*/*Z* isomerism. Dhb is the simplest α-dhAA that exhibits *E*/*Z* isomerism. The *Z*-isomer of Dhb (*Z*-Dhb) is sterically favourable and is present in the majority of naturally occurring peptides containing Dhb residues. Dha and *Z*-Dhb motifs are commonly found in ribosomally synthesized and post-translationally modified peptides (RiPPs). In the last decade, the formation of Dha and Dhb motifs in RiPPs has been extensively investigated, which will be briefly discussed in this review. The formation of other dhAA residues in natural products (NPs) is, however, less understood. In this review, we will discuss recent advances in the biosynthesis of peptidyl NPs containing unusual dhAA residues and cryptic dhAA residues. The proposed biosynthetic pathways of these natural products will also be discussed.

## Abstract 2

In this review, we will discuss recent advances in the biosynthesis of peptidyl NPs containing unusual dhAA residues and cryptic dhAA residues. The proposed biosynthetic pathways of these natural products will also be discussed.

## Graphical abstract

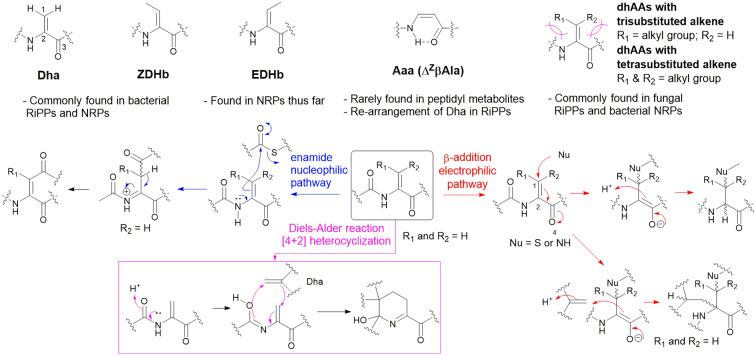

## 1 Introduction

### 1.1 Naturally occurring dehydroamino acid residues

**Fig. 1:**
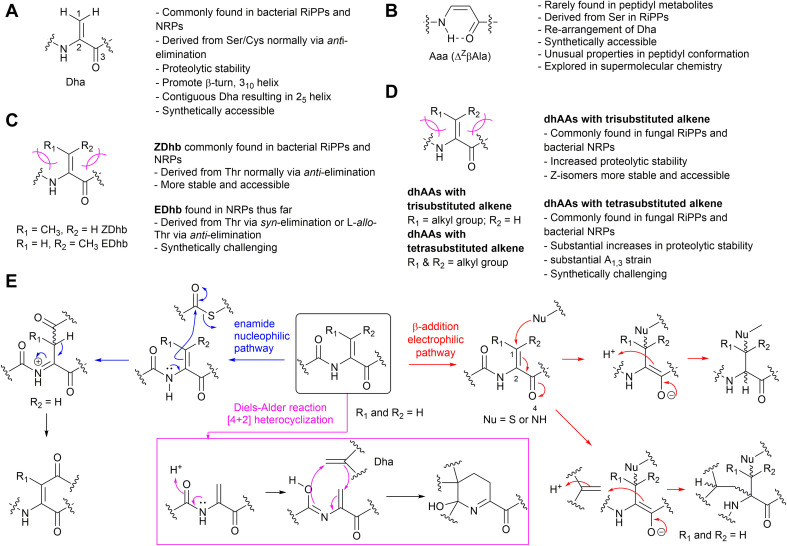
The structures and general information of naturally occurring dhAAs and their involvements in various reactions discovered during the maturation of peptidyl metabolites. (A) Dha residue. (B) Aaa (Δ^Z^βAla) residue. (C) Dhb isomers. (D) Other dhAA residues. (E) Three types of reactions involving transient dhAA residues found so far.

Naturally occurring α,β-hehydroamino acids (dhAAs) (Fig. 1) are unsaturated noncanonical amino acids. Their main structural feature comprises an alkene motif, which in most cases is placed between the α-carbon (C-α) of the carbonyl and β-carbon (C-β) of the amino acid to give α,β-dehydro-amino acids, which will be discussed in this review.

Unlike standard amino acids, which display asymmetry at the C-α position, the alkene motif in dhAAs constricts the position of the β-substituents, which leads to the appearance of isomers *Z* and *E*. The presence of an α,β-double bond in dhAAs also results in a distinctive electronic architecture compared to the standard analogues. The lone-pair electrons at the adjacent nitrogen readily conjugate with the double bond of the alkene. As such, the free-amino forms of most dhAAs at the N-termini of peptides are unstable, readily hydrolysing in mildly acidic aqueous solution to liberate ammonia and an α-keto acid. On the other hand, the π-electron conjugation between the α,β-double bond and flanking electron-withdrawing carbonyl also makes dhAAs exhibit electron-poor features, making dhAAs competent partners for a variety of chemical transformations. For example, dhAAs are fairly reactive Michael-like acceptors that react readily with thiols and amines^1^ but are less reactive toward oxygen-containing nucleophiles. Notably, dhAAs have been implicated in other additions, such as radical additions, transition-metal-catalysed cross-coupling reactions, and cycloadditions.^2^ The synthetic potential of dhAAs is now being extensively explored in biological contexts due to the identification of milder and more selective activating reagents that work at ambient temperature and in aqueous media. This can be exemplified by photochemical bioconjugation to dhAAs under mild conditions, which has recently emerged.^3^ Visible-light photoredox catalysis on dhAA-containing peptides/proteins offers high reactivity and selectivity, fast kinetics and good functional-group tolerance, and results in new functionalities, and chemistry that nature is yet to explore, within NP scaffolds. The chemical space available through dhAA-modification has also spurred its use in precise engineering of drug-like peptidyl molecules at the late stage of drug discovery and new applications in chemical biology.

Among <40 dhAAs found in the natural product (NP) inventory, dehydroalanine (Dha) and dehydrobutyrine (Dhb) are the most abundant.^4^ Dha is the simplest dhAA without geometrical isomers, while its re-arranged isomer, 3-aminoacrylic acid (Aaa or ΔβAla), is the simplest dehydro-β-amino acid, or β-enamino acid, and displays *E*/*Z* isomerism. Dhb is the simplest α-dhAA that exhibits *E*/*Z* isomerism. Due to their reactivities, dhAAs have been found as part of cyclic motifs in structurally diverse NPs *via* three different chemical pathways of either 1,4-Michael addition, nucleophilic substitution or [4+2] Diels–Alder reactions (Fig. 1E).

### 1.2 The presence of dhAA residues in peptide designs and materials

The α,β-double bond in dhAAs has a profound effect on the conformation of the whole peptide molecule. For example, dhAA residues have a rigidifying effect on the peptide backbone, which can increase peptide–receptor affinity by reducing the entropic costs of binding.^5^ Another feature of dhAA-containing peptides is increased stabilities to degradative enzymes,^5^ which has led to synthetic enzyme inhibitors that act as nonhydrolyzable substrate mimics. dhAA residues sometimes occur in enzyme active sites and in naturally occurring enzyme inhibitors, where they may serve as electrophiles in nucleophilic addition reactions. These features have generated interest in dhAA-containing peptide-based therapeutic agents.^2^

**Fig. 2:**
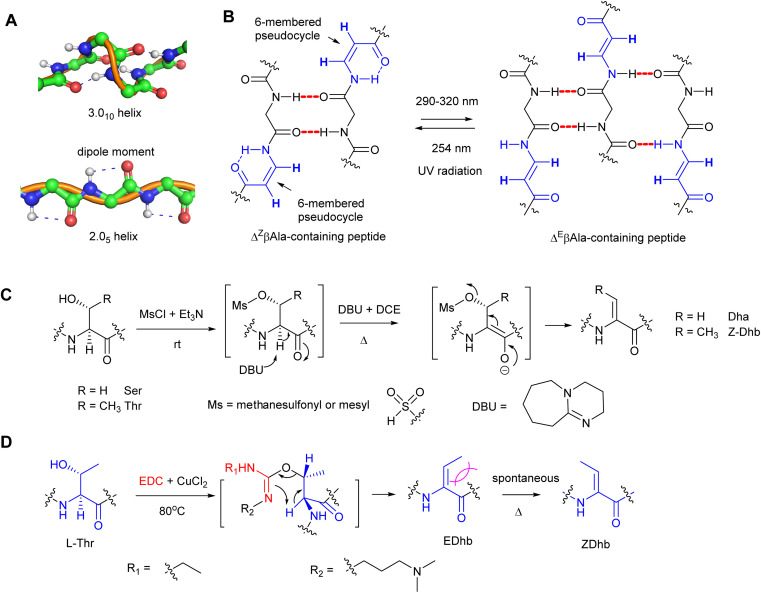
The influences of peptide configurations in the presence of dhAA residues and common synthetic methods to generate Dha/Dhb residues in peptide chemistry. (A) The normal 3.0_10_-helix in proteins (top) and the unusual 2.0_5_-helix when peptidyl foldamers contain contiguous Dha residues (bottom). (B) The conformational changes of *Z*- and *E*-isomers when ΔβAla-containing peptides are treated with UV irradiation. (C) A common synthetic method in peptide chemistry to provide a Dha/*Z*-Dhb residue *via* an overall *anti*-elimination pathway. (D) A common synthetic method in peptide chemistry to provide an *E*-Dhb residue *via* an overall *syn*-elimination pathway.

dhAAs are useful tools to build new 3D-structures that can be exploited in supramolecular chemistry. For example, Dha is known to preferentially adopt the fully-extended conformation to form a 2.0_5_-helix because it is characterized by 2 residues per turn and stabilized by strong dipole moments encompassing 5-membered pseudo-cycles (Fig. 2A).^6^ In this peculiar type of helix, the rotation per residue along the helix axis is exactly 180°.^7,8^ As a result, stretches of Dha arrange as flat foldamers.^9^ On the other hand, dhAAs with a bulkier substituent at the β-carbon, such as Dhb, promote the formation of β-turns or 3.0_10_ helices in peptide structures.^10^

Recent studies revealed that peptidyl foldamers containing β-enamino acid units, such as 3-aminoacrylic acid (ΔβAla), display interesting conformational, electronic, and supramolecular aggregation properties that can be modulated by selective *E*–*Z* photoisomerization.^11–14^ The conformation of the Δ^Z^βAla moiety in the peptide allowed formation of an N–H⋯O=C intra-residue H-bond, which closes a 6-atom pseudo-cycle (Fig. 2B), making a kink in the overall shape of the foldamer. Its packing modes were layers of antiparallel molecules. This was not the case for the Δ^E^βAla-containing peptide, where no pseudo-cycle is formed and each molecule is connected to its neighbours by four intermolecular H-bonds, two on each side, giving rise to a flattened β-sheet (Fig. 2B). When treated with irradiation at 290–320 nm, the Δ^Z^βAla-containing peptide was quantitatively photoconverted to its (*E*)-isomer, which, upon irradiation at 254 nm, could be converted back to the (*Z*)-isomer. Transmission electron microscopy (TEM) analysis indicated that the increasing (*E*)/(*Z*) molecular ratio resulting from the photoconversion gives rise to the formation of fibres of increasing size.

### 1.3 Construction of dhAA motifs in peptidyl molecules

Since Dha and Dhb are the predominant dhAAs found in peptide natural products, a number of synthetic methods have been developed, among which β-elimination would be the most straightforward approach to access Dha and Dhb residues. These methods involve activation of the β-hydroxy group of the precursor amino acids and subsequent β-elimination under alkaline conditions.

Compared to the simple Dha, stereoselective construction of *E*- and *Z*-isomers of Dhb is required because *Z*- and *E*-conformations in Dhb-containing peptides determine the bioactivities of the products.^15–17^ Several practical routes to *Z*- and *E*-α,β-dhAAs have been developed. For example, a stereoselective *anti*-elimination using an MsCl/DBU dehydration system to generate *Z*-Dhb was developed (Fig. 2C). However, the high 1,3-allylic strain at an *E*-Dhb residue results in thermodynamic instability in comparison to its *Z*-counterpart, making its selective synthesis highly challenging.^17^ Although the *E*-isomer can be generated from an l-*allo*-Thr derivative,^15^l-*allo*-Thr is expensive (∼£800 per g from Sigma), preventing this method from being widely applied. An alternative approach to *E*-Dhb was introduced by using 1-ethyl-3-(3-dimethylaminopropyl) carbodiimide (EDC) in the presence of CuCl_2_ to generate *E*-Dhb-containing peptides *via* the *syn*-elimination pathway^18^ (Fig. 2D). A short reaction time was found to be important for high *E*-selectivity because the produced *E*-Dhb can slowly isomerize into the more stable *Z*-isomer at elevated temperature,^18^ resulting in a mixture of *E*- and *Z*-isomers. Synthetic methods for other dhAA constructions can be found in the comprehensive review.^19^

### 1.4 Purpose of the review

This review surveys dhAA-containing peptidyl natural products isolated from bacteria, fungi and plants with antimicrobial and anticancer activities, highlighting the biosynthetic origins of these modified non-canonical amino acid residues. These peptides include characterized ribosomally synthesised and post-translationally modified peptides (RiPPs), non-ribosomal peptides (NRPs) and peptides with unknown biosynthetic origins. Selected characterized pathways involved in their biosynthesis and the key enzymes that catalyse the dehydration reaction are also summarised.

Bacterial non-ribosomal peptides (NRPs) and their biosynthesis are the focus of this review. The period of 2015–2023 saw our improved understanding of how the catalytic domains of non-ribosomal peptide synthetases (NRPSs) and associated tailoring enzymes process various types of chemical transformations to provide dhAA residues and motifs involved in transient dhAAs in the growing peptidyl chains or during the maturation processes. It is worth noting that, although the origins of Dha/Dhb from selected NRPs have been determined biochemically, other dhAAs have remained uncharacterized in the corresponding biosynthetic pathways of these NRPs. Bioinformatic and phylogenetic analysis using current knowledge would shed light on how these dhAAs are produced. This will also be discussed in this review.

Bacterial RiPPs represent a growing group of structurally diverse peptidyl natural products, many of which contain multiple Dha/Dhb moieties and motifs containing transient Dha/Dhb residues. The biosynthesis and mechanisms of how these dhAAs are synthesized have been extensively investigated. The readers are referred to the reviews led by van der Donk.^20,21^ Hence, in this review we aim to update and complement previous synopses and cover only these newly identified unusual dhAAs and motifs involved in transient Dha/Dhb that have not been covered in the aforementioned review.^21^

Fungi are among the prolific producers of bioactive peptides, and thus they are a topic of this review. Although originally thought to be non-ribosomal peptides due to the presence of a variety of non-proteinogenic amino acid residues, many fungal peptides have now been characterized to be RiPPs. Some of these fungal RiPPs have also been covered in the aforementioned review.^21^ However, we include the recent biosynthetic understanding of fungal RiPPs and their associated dhAA formation not mentioned in the above review.^21^

Plants produce a variety of bioactive cyclic peptides, some of which are likely to have ribosomal origins. It is rather surprising to observe that the presence of dhAA residues in plant peptides is exceedingly rare. The the formation of these residues is poorly understood because the study of biosynthesis of plant metabolites is still in its infancy.

We also explore dhAA-containing peptides from marine animals. These organisms have been shown to produce structurally diverse dhAA-containing peptides with potent bioactivities. Although it has been long speculated that these NPs originate from microbial symbionts in these animals, the detailed biosynthetic characterization of these peptides and associated dhAA residues has not been disclosed.

Finally, it is not our intention to include dhAAs linked with other residues, such as 5-membered heterocycles, and other modifications such as *N*-methylation of dhAA residues. In the case of dhAA with 5-membered heterocycles, it is likely that the dhAA is formed first, followed by heterocyclization of the dhAA carbonyl and the adjacent amino acid to form such motifs. The latter would be an *N*-methylation followed by dhAA formation.

## 2 dhAAs in ribosomal peptides and proteins

### 2.1 dhAA residues in bacterial RiPPs

**Fig. 3:**
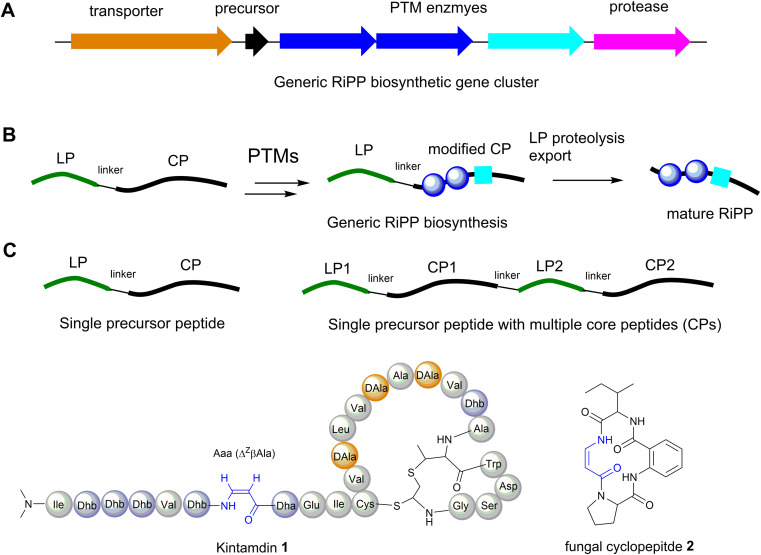
(A) Generic RiPP biosynthetic gene cluster (BGC). (B) A general scheme of RiPP biosynthesis. (C) Precursor peptide containing only one core peptide and precursor peptide containing multiple core peptides found in RiPP BGCs. (D) Structure of cyclopeptides containing β-enamino acid residues, highlighted in blue. The amino acid residues are colour coded. Proteinogenic amino acids: light grey; Dha/Dhb: light blue; bicyclic methyl-amino-bithionin (MAbi): bold red; d-amino acids: orange.

The majority of ribosomally synthesised and post-translationally modified peptides (RiPPs) are synthesized from precursor peptides, encoded by a structural gene (Fig. 3A). The precursor peptide generally includes an N-terminal leader peptide (LP), which often dictates the binding of modification enzymes, and a C-terminal core peptide (CP), which is enzymatically modified and then released to form the advanced biosynthetic intermediate or final product (Fig. 3B). In most cases studied, a precursor peptide contains a single CP. The precursor peptides of fungal and plant RiPPs^21^ and some bacterial RiPP families feature multiple CP motifs (Fig. 3C).^22^

Although RiPPs exploit only the 20 proteinogenic amino acids, extensive post-translational modifications (PTMs) increase their structural diversity.^21^ They have been discovered across all three domains of life. Among these structurally diverse peptides, bacterial RiPPs are the most well-established, simply because genes of these biosynthetic pathways are clustered, which would allow ready interrogation of the functions of these genes *in vivo* or *in vitro*. Dha and *Z*-Dhb residues are commonly found in RiPPs, and are derived from serine/cysteine and threonine, respectively. To date, the observed configuration of Dhb moieties requires the *anti*-elimination of activated Thr residues to generate a *Z*-Dhb. Comprehensive information on chemical classes of bacterial RiPPs can be found in a recent review.^21^

Most RiPPs discovered so far only contain α-amino acid residues. However, recent studies indicated that β-amino acid residues can be formed in the RiPP pathways, including the α-keto-β-amino residue in spliceotides widely distributed in cyanobacteria^23,24^ and the isoaspartate residue in some class I lanthipeptides.^25^ We have recently reported the discovery of an unusual RiPP called kintamdin 1 from an environmental isolate of *Streptomyces* sp. RK44 (Fig. 3D).^26^ Apart from proteinogenic amino acids and post-translationally modified residues such as Dha, Dhb and d-Ala, which have been observed in other RiPPs, the peptide contains an unusual β-enamino acid residue, (*Z*)-3-amino-acrylic acid (Aaa), which, to the best of our knowledge, has not been reported in any other RiPPs.^26^ The existence of such an amino acid residue is also extremely rare in the natural product inventory. Only one cyclopeptide 2, isolated from a marine gut fungus, *Aspergillus flavipes*, from *Ligia oceanica*, contains the Aaa residue (Fig. 3D).^27^ Considering that the 2-amino-benzoic acid residue is not a proteinogenic amino acid, it is likely that 2 is of non-ribosomal origin.

### 2.2 Motifs containing transient dhAA residues in bacterial RiPPs

**Fig. 4:**
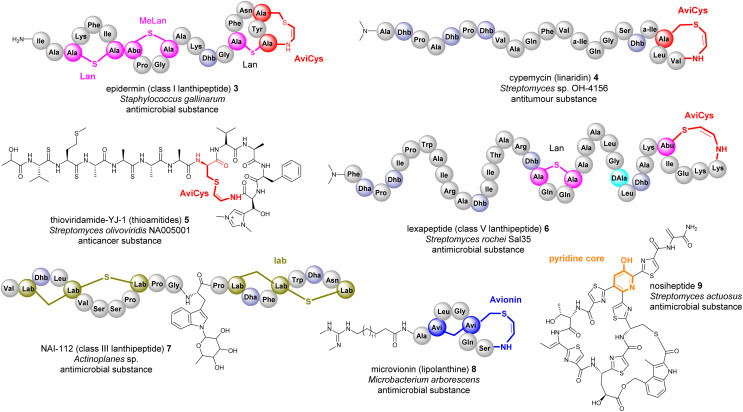
Representative structures of bacterial RiPPs containing Lan, AviCys, Lab, avionin and pyridine motifs that are derived from transient Dha residues. Amino acid residues are colour coded. Proteinogenic amino acids: light grey; Dha/Dhb: light blue; Lan: bold pink; AviCys: bold red; Lab: bold green; avionin: bold blue; pyridine: bold orange.

Many bacterial RiPPs contain a range of cyclic motifs that originate from transient dhAA residues. When Dha and cysteine or decarboxylated cysteine residues are involved, dedicated cyclization enzymes provide noncanonical thioether amino acid residues, such as lanthionine (Lan) and 2-aminovinyl-cysteine (AviCys) (Fig. 4A). The characterized Lan motifs in lanthipeptides contain (2*S*,6*R*) configurations or dl-Lan (d refers to d-Ala and l refers to l-Cys) (Fig. 4). The AviCys moieties in epidermin 3 and cypemycin 4 were determined to be *S*-[(*Z*)-2-aminovinyl]-d-cysteine. When a second Dha motif is involved, the triamino triacid, labionin (Lab) and AviCys-labionin (avionin, a decarboxylated analogue of Lab), can be formed in NAI-112 7, a class III lanthipeptide, and microviridin 8, a lipolanthine, respectively (Fig. 4). The stereochemistry of Lab reported thus far is (*S*,*S*,*R*) in the l-Ala-d-Ala-l-Cys sequence. When two Dha residues together with an adjacent amino acid residue are involved, a six-membered nitrogen containing motif, pyridine, dehydropiperidine or piperidine, can be generated through an intramolecular Diels–Alder [4+2] cyclization pathway. These cyclic motifs are the key feature of a large group of RiPPs called pyritides, such as nosiheptide 9 (ref. 21) (Fig. 4).

**Fig. 5:**
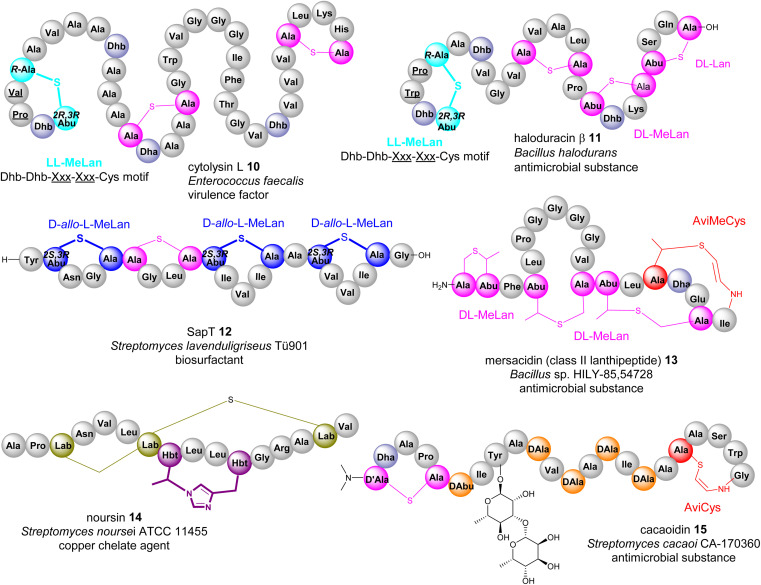
Representative structures of bacterial RiPPs containing various transient Dhb residues. Amino acid residues are colour coded. Proteinogenic amino acids: light grey; Dha/Dhb: light blue; Lan/dl-MeLan: pink; ll-MeLan: cyan, bold; d-*allo*-l-MeLan: blue; AviCys/AviMeCys: red; Lab: green; Hbt: purple; d-amino acids: orange.

If a Dhb residue is involved in a cyclization reaction with l-Cys, the stereochemistry of the cyclic motif, methyllanthionine (MeLan), can be complicated. Three diastereoisomers, (2*S*,3*S*,6*R*) (dl-MeLan), (2*R*,3*R*,6*R*) (ll-MeLan) and (2*S*,3*R*,6*R*) (d-*allo*-l-MeLan), have been found in RiPPs.^28^ Currently there is no report to indicate the presence of l-*allo*-l-MeLan. While most characterized lanthipeptides contain dl-MeLan moieties, the other two diastereoisomers are rather unusual (Fig. 5). Two class II lanthipeptides, cytolysin 10 from *Enterococcus faecalis* and haloduracin 11 from *Bacillus halodurans*, were found to contain a mixture of dl-Lan and ll-MeLan (Fig. 5).^28^ More recently, the existence of d-*allo*-l-MeLan (2*S*,3*R*,6*R*) was found in SapT 12, a class I lanthipeptide, which was shown to contain one dl-Lan but three d-*allo*-l-MeLan residues through a combination of *E. coli* gene co-expression and comparison of chemically derivatized MeLan fragments of the mature peptide with synthetic MeLan fragment diastereoisomers (Fig. 5).^29^

Like Dha, transient Dhb residues have been found to react with decarboxylated Cys residues to provide AviMeCys during the biosynthesis of RiPPs. Unlike the three diastereoisomers of MeLan found in RiPPs, only *S*-[*Z*]-2-aminovinyl-(3*S*)-3-methyl-d-cysteine ((2*S*,3*S*)-AviMeCys) residues have been characterized so far in a few RiPPs such as mersacidin 13 (Fig. 5).^30^ There has been no report of transient Dhbs being involved in the formation of Lab or Avionin residues thus far.

Kintamdin 1 also contains an unprecedented structural element, bicyclic methyl-amino-bithionin (MAbi), a motif that was first found in natural products.^26^ It is likely that this cyclic motif results from two rounds of addition reactions among a transient *Z*-Dhb-22 residue, Cys-11 in the middle of the peptidyl chain, and the C-terminal decarboxylated Cys-27 to provide four chiral centres at positions 11, 22α, 22β and 27 (amino acid residue numbers in kintamdin) (Fig. 3). Although computational modelling analysis combined with interpretation of long-ranged NOE correlations suggested that the MAbi contains 11*R*, 22α*S*, 22β*S* and 27*S* stereogenic centres, the exact configurations require experimental verification. The timing of these two cyclization events also remains to be determined.

Transient dhAAs can also react with a nitrogenous donor in RiPPs. Noursin 14, a new class III lanthipeptide, was discovered in *Streptomyces noursei* ATCC 11455, the structural element of which contains an unusual His-butyrine (Hbt) crosslink between transient Dhb 8 and His 15 (Fig. 5). Computational analysis combined with NOESY NMR interpretation allowed the stereochemical assignment of Hbt as likely (*R*)-Cα-(*S*)-Cβ in the butyrine moiety. This is the first example of the crosslink between dhAA and a nitrogenous donor in RiPPs.

Although generated from l-amino acids, some bacterial RiPPs, such as lanaridins/lanthidins/lanthipeptides, also contain d-amino acid residues. The predominant d-amino acid in RiPPs is d-Ala, which is derived from a transient Dha through a stereospecific reduction reaction. Few RiPPs, such as cacaoidin 15 (Fig. 5), have been found to contain d-Abu residues so far, which are likely to be derived from transient *Z*-Dhbs.^31–33^

### 2.3 dhAAs in fungal and plant RiPPs

Although fungi and plants contribute many peptidyl metabolites, the biosynthesis of these metabolites is poorly understood because, unlike the clustered biosynthetic gene clusters in bacterial genomes, biosynthetic genes are normally scattered in the genomes of fungi and plants, thus making it extremely difficult to correlate the chemical structures with the biosynthetic origins. Therefore, whether many fungal and plant peptides are of ribosomal or nonribosomal origin remains to be determined. As such, only experimentally confirmed RiPPs will be discussed in this section.

**Fig. 6:**
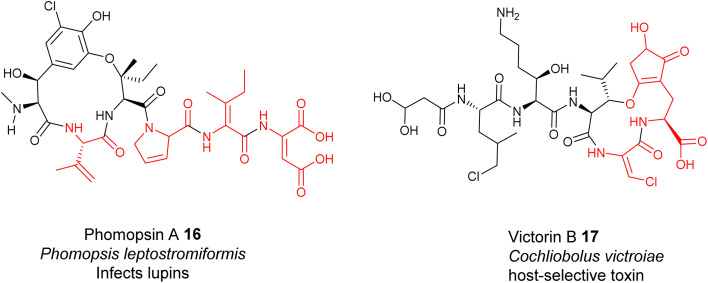
Representative structures of fungal RiPPs with dhAA residues: phomopsin A 16 and victorin B 17.

Unlike the Dha/Dhb predominance in bacterial RiPPs, dhAAs in fungal RiPPs display greater structural diversity, suggesting that enzymes responsible for these dhAA residues may be completely different from those in bacteria. This can be exemplified by *E*-dehydroIle (EΔIle), *E*-dehydroAsp (EΔAsp), β,γ-dehydroPro (β,γ-ΔPro), and β,γ-dehydroVal (β,γ-ΔVal) in the mycotoxins, phomopsins 16, produced by the pathogenic ascomycetes *Phomopsis leptostromiformis* that infects lupins^34,35^ (Fig. 6). Others contain heavily modified dhAA residues, such as *E*-3-chloro-Dha in the victorins, 17, produced by the necrotrophic fungal pathogen *Cochliobolus victroiae*^36^ (Fig. 6).

Plants are prolific producers of cyclic, peptidyl natural products, many of which are RiPPs. However, dhAA-containing plant peptides are exceedingly rare. Their bio-origins remain to be determined and will be discussed in Section 4.

### 2.4 Enzymes responsible for the formation of dhAA residues in bacteria

**Fig. 7:**
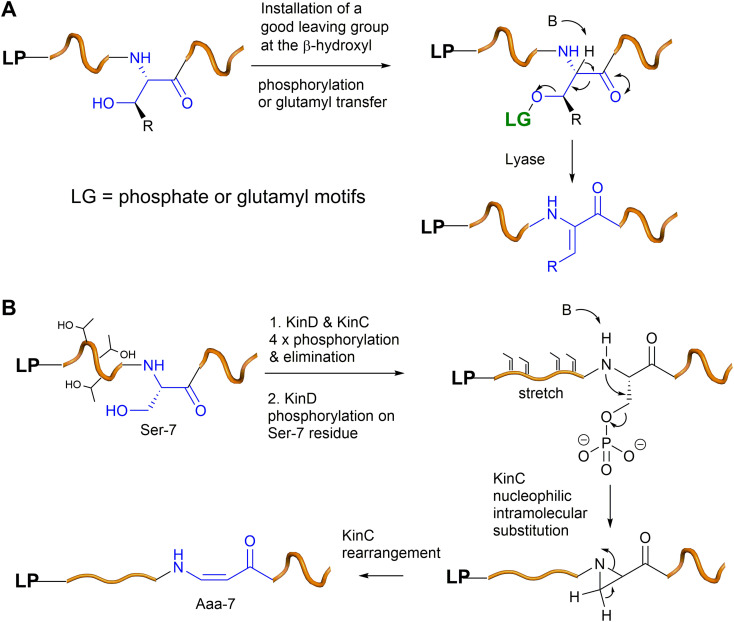
(A) A general scheme of Dha/*Z*-Dhb formation in bacterial RiPPs. (B) A proposed mechanism of the Aaa-7 formation in kintamdin.

The enzymes involved in dehydration of Ser and Thr to provide Dha and *Z*-Dhb in lanthipeptides have been extensively studied *via* the seminal works of the van der Donk group. Generally, the β-hydroxyl group of Ser and Thr residues in the lanthipeptides need to be installed with a good leaving group (*i.e.*, phosphate or Glu) *via* dedicated enzymes (*i.e.*, kinases or tRNA transferases), followed by the actions of lyases to provide Dha and *Z*-Dhb (Fig. 7A), akin to the preparation of Dha and *Z*-Dhb in organic reactions, as shown in Fig. 2C. One exception was found in cypemycin, where the Dha-19 residue is derived from Cys-19 *via* direct dethiolation by the corresponding lyase.^30,37^

In the last five years, a group of kinases and lyases has been found to be involved in the formation of other groups of RiPPs, including cypemycin 4 from the linaridins, thioviridamide 5 from the thioamitides, and lexapeptide 6 as a class V lanthipeptide, as shown in Fig. 4.^31,38–42^ These PTM enzymes, responsible for phosphorylation and elimination, share very low amino acid sequence similarity to the ones involved in the formation of lanthipeptides I–IV, but the underlying mechanisms for the dehydration of the β-hydroxyl group of Ser/Thr are identical (Fig. 7A). The biosynthesis of kintamdin 1 was partially characterized through *E. coli* gene co-expression approaches. It was found that the kinase homologue KinD and the HopA1-like enzyme KinC are responsible for Ser/Thr dehydration to install dhAAs in 1. It is highly likely that KinCD is processive in the phosphorylation and elimination of one amino acid at a time, starting from the N-terminus of the core peptide (CP) (Fig. 7B). Interestingly, the N-terminal of the CP of KinA is enriched with four Thr (Thr-2, 3, 4 and 6) residues that are converted into corresponding Dhb moieties, which have a profound impact on the proceeding CP. It is likely that substrate-controlled dehydration occurs after the first four Thr residues are dehydrated, resulting in a fully extended CP. Such conformation changes are likely to play an important role in KinD-catalysed phosphorylation. Changing all four of these Thr residues to Ala in the CP causes less efficiency for the phosphorylation of Ser-7 in the proceeding CP. The stretching of the CP is likely to affect KinC activity, causing the abstraction of the NH hydrogen of the amide between Dhb-6 and phosphoSer-7, followed by a nucleophilic attack at the β-carbon of phosphoSer-7 to likely yield an aziridine intermediate (Fig. 7B). Such a re-arrangement is akin to the conversion of l-Thr in peptides into an aziridine motif using Mitsunobu reagents.^43,44^ Subsequently, KinC could further catalyse a ring opening of the aziridine-containing intermediate to provide Aaa-7, similar to a chemical precedent.^45^ Once Aaa-7 is formed, the enzyme pair, KinCD, catalyses the remaining 6 rounds of dehydration events.

**Fig. 8:**
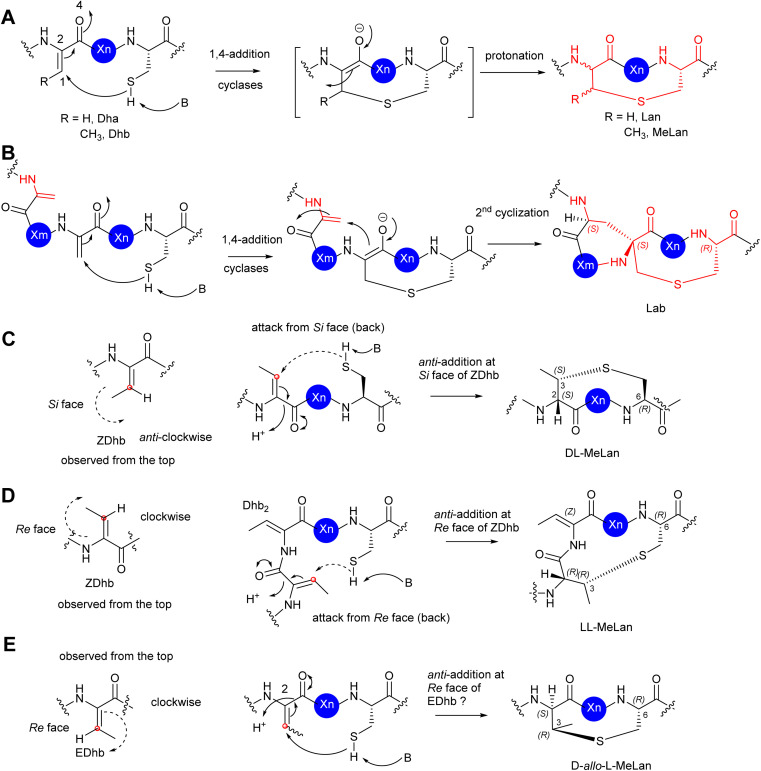
(A) The mechanism of the formation of Lan/MeLan in bacterial RiPPs from a transient Dha/*Z*-Dhb, respectively, and a Cys residue. (B) The mechanism of the formation of Lab from a transient Dha and a Cys residue. (C) The proposed formation of dl-MeLan. The nucleophilic attack of a Cys residue results in 1,4-addition to the *Si* face of a transient *Z*-Dhb. The overall outcome is an *anti*-addition reaction. (D) The proposed formation of ll-MeLan. The nucleophilic attack of a Cys residue results in 1,4-addition to the *Re* face of a transient *Z*-Dhb. The overall outcome is an *anti*-addition reaction. (E) The proposed formation of dl-*allo*-MeLan. The nucleophilic attack of a Cys residue results in 1,4-addition to the *Re* face of a possible transient *E*-Dhb. The overall outcome is an *anti*-addition reaction. The red dots represent the carbon centre of the alkene face. The dash arrows represent attack from the back. The solid arrows represent attack from the top.

In the cases of cyclic motifs, such as Lan/MeLan and Lab, the biosynthetic pathways recruit either a Zn^2+^-dependent (LanC for class I, LanM for class II, and LanL for class IV lanthipeptides) or metal-free cyclase (LanKC for class III lanthipeptides) to facilitate the ring closure. In most cases of Lan/MeLan/Lab residues, the corresponding cyclases of lanthipeptides catalyse the addition between the thiol group of Cys and the *Si* face of the alkene motifs of Dha/*Z*-Dhb *via* an *anti*-addition pathway to give dl-Lan or dl-MeLan, respectively^28^ (Fig. 8A–C). This is not the case for cytolysin 10 and haloduracin 11, which contain dl-Lan and ll-MeLan resulting from the same cyclases encoded in the corresponding BGCs.^46^ In these special cases, while the cyclases provide the common dl-Lan between the corresponding Dha and Cys on the *Si* face of Dha, ll-MeLan residues result from the Dhb_1_-Dhb_2_-Xxx-Xxx-Cys sequences where the cyclizations occur between Dhb_1_ and Cys (Fig. 8D). Changing Dhb_2_ to Ala in the precursor peptides instead results in the formation of dl-MeLan, suggesting a substrate-control hypothesis.^47^ Computational analysis suggested that a conformational preference caused by these two contiguous Dhb residues is unfavourable for *anti*-addition on the *Si* faces of the alkene motifs. However, the cyclization enzymes can still activate the Cys residues, but only the substrate-controlled *Re* face of the alkene is available for the following *anti*-addition reactions to provide ll-MeLan (Fig. 8D).^47^

The formation of d-*allo*-l-MeLan in SapT 12 is intriguing. A sequence similarity network suggested that SptB_b_ in the pathway of 12 is a unique member of the glutamyl lyase family that has a different arrangement of amino acid residues in its active site compared to other characterized glutamyl lyases, hinting at a different catalytic activity compared to other common ones.^29^ Considering the high sequence similarity of the cyclase, SptC, to others for class I lanthipeptides, it was deduced that the cyclization would follow the *anti*-addition pathway on the corresponding alkene motif. If the Dhbs are in the *Z* conformation, such a reaction must follow a *syn*-addition pathway to form d-*allo*-l-MeLan, which is unlikely to be catalysed by SptC. As such, an *anti*-addition pathway between l-Cys and the *Re*-faces of *E*-Dhb residues was proposed (Fig. 8E). If it were true, this would be the first example that RiPPs can generate transient *E*-Dhb residues, which requires a group of uncommon lyases, such as SptB_b_, to facilitate the *syn*-elimination reaction on the activated l-Thrs.^29^ Further investigation is needed to examine this hypothesis.

**Fig. 9:**
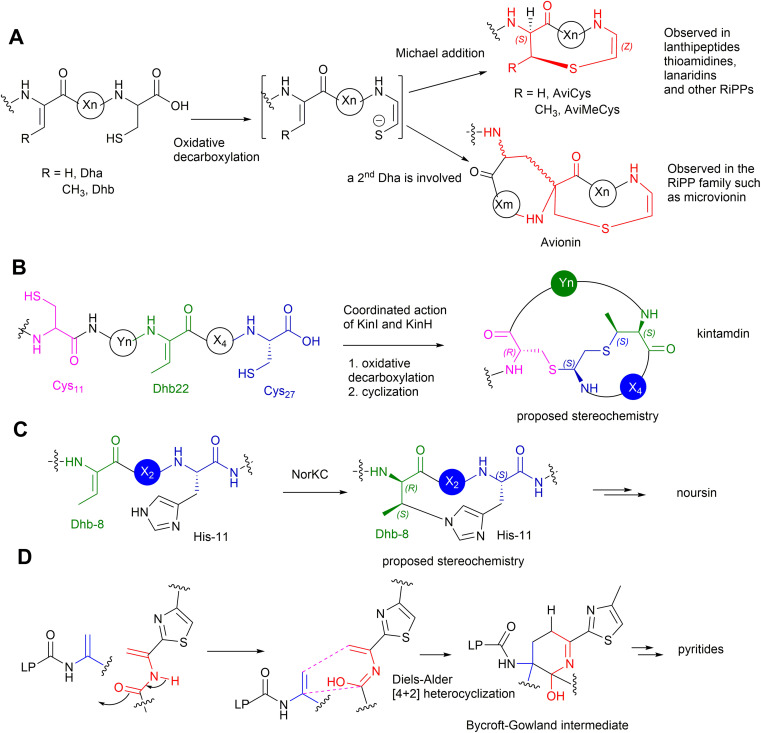
(A) The mechanism of formation of cyclic AviCys/AviMeCys and Avionin residues in bacterial RiPPs from a transient Dha/*Z*-Dhb, respectively, and a decarboxylated Cys residue. (B) The proposed mechanism of the formation of the bicyclic MAbi residue from the transient Dhb-22, the Cys-11 and the decarboxylated Cys-27 residues. (C) The proposed formation of cyclic Hbt from the transient Dhb-8 and His-11 residues. (D) The proposed formation of pyridine/dehydropiperidine/piperidine *via* an intramolecular Diels–Alder [4 + 2] cyclization between two transient Dha residues to generate a Bycroft–Gowland intermediate, followed by modification (*i.e.* dehydration/reduction) to provide the desirable six-membered nitrogenous motif.

The PTMs involved in the formation of AviCys/AviMeCys residues proceed *via* an oxidative decarboxylation of the C-terminal Cys to provide a reactive thioenol nucleophile, followed by addition of a Dha or Dhb residue to provide AviCys or AviMeCys, respectively (Fig. 9A). The best examples are the FMN-dependent EpiD in the biosynthesis of epidermin 3 and the FAD-dependent MrsD in the biosynthesis of mersacidin 13.^31–33^ Both enzymes alone can catalyse oxidative decarboxylation, followed by cyclization. Although homologues of EpiD and MrsD were found for linaridins, and thioamitides, *in vitro* enzyme assays using either recombinant precursor peptides or synthetic substrate mimics, although displaying decarboxylation activities, failed to provide any cyclic products.^48,49^ The formation of AviCys residues in 3 and 6 would actually require enzyme partners, which are likely to be inactive kinase-like proteins, to coordinate with the corresponding decarboxylases.^38,48^ A similar case has also been also found in MAbi formation during the biosynthesis of 1, where the flavoprotein KinI requires the presence of kinase-like KinH to facilitate the oxidative decarboxylation and subsequent bicyclic crosslinking in *E coli* gene co-expression experiments^26^ (Fig. 9B). Changing Cys-11 to Ala also results in abolishment of cyclic peptides, indicating that the presence of Cys-11 is essential for the bicyclization events. The factors that determine MAbi/Lab/Avionin formations remain to be determined.

Biochemical analysis has demonstrated that the dedicated cyclase, NorKC, in the pathway of 14 catalyses the formation of the (3*S*,7*S*,15*R*)-Lab motif first, followed by the crosslink between His-11 and Dhb-8 to provide the Hbt residue (Fig. 9C).^50^ It is likely that the constraints generated by the Lab motif in the peptidyl intermediates as they are processed contribute the increased reactivity of Dhb-8. Phylogenetic analysis has indicated that NorKC and its homologues form a separate cluster distinct from LanKC enzymes for the typical class III lanthipeptides.^50^

The six-membered, nitrogenous heterocycles featured in pyritides are fashioned from two transient Dha residues of the linear peptide precursors in a single enzymatic biotransformation.^51^ The cycloaddition initiates with the tautomerization of one Dha-adjacent amide to an iminol as a suitable diene, followed by a [4+2] Diels–Alder reaction with another Dha to yield a cyclic hemiaminal intermediate, called a Bycroft-Gowland intermediate, which serves as a key branching point for pyritides containing various forms of pyridine motifs (Fig. 9D).^52^ Recent studies demonstrated that a tyrosine residue in the active site of a pyritide synthase in the thiopeptide pathway facilitates the final aromatization step of pyridine formation.^53^

Many RiPPs contain d-amino acid residues. Recent studies demonstrated that a F420-dependent reductase in the biosynthesis of lexapeptide 6 catalyses an iterative biotransformation, changing the corresponding Dha residues to d-Ala in a stereospecific manner.^54^ It is likely that similar enzymes would be responsible for the formation of d-Abu residues in other RiPPs.

Unlike the well-studied dhAAs in bacterial RiPPs, the entries of dhAA residues into fungal and plant RiPPs are poorly understood. Although it has been known since 2007 that RiPPs are produced by strains from fungal phyla, the majority of fungal peptidyl metabolites were originally thought to be non-ribosomal peptides, because they contain many non-canonical amino acid residues. In the last ten years, advances in transcriptome analysis coupled with accurate genome annotation have allowed real appreciation that fungal RiPP pathways contain completely different biosynthetic PTM enzymes for providing dhAA residues. For example, it was reported that the cyclic hexapeptide phomopsin A 16 from the pathogenic ascomycetes *Phomopsis leptostromiformis* features a set of non-proteinogenic dhAAs that have a ribosomal origin.^35^ Interestingly, the BGC contains 15 conserved genes, 5 of which encode domain of unknown function (DUF)-3328 (Pfam ID: PF11807; InterPro family: IPR021765), a family of proteins only found in eukaryotes. Members of DUF3328 have been found to be involved in a range of oxidative transformations, such as oxidative cyclization, chlorination, hydroxylation and transacylation, during the biosynthesis of various fungal peptidyl NPs.^36,55–57^ More recently, a series of gene inactivations were performed in the phomopsin-producing strain.^35^ Interpretation of accumulated intermediates among the genetic variants strongly suggested that three DUF3328 homologues, PhomYc, PhomYd and PhomYe, are responsible for the formation of ΔIle, ΔAsp and β,γ-ΔPro residues in phomopsins, respectively. However, the genes responsible for the formation of β,γ-ΔVal remain to be determined.

### 2.5 dhAAs in proteins

dhAA residues, although rarely found in proteins, play important biological roles in protein functions.^58^ For example, during infection processes, pathogenic bacteria can deliver effector proteins, such as phosphothreonine lyases, into host cells to modulate signalling pathways. These proteins belong to the HopA1 protein superfamily and have the unique ability to catalyse β-elimination of phosphorylated Thr (pThr) or Ser (pSer) residues to provide Dhb or Dha, respectively,^59,60^ with a phosphorylation preference for Thr residues.^60^ It has been established that, during bacterial infection, the enzymes attenuate the innate immune response by mainly deactivating mitogen-activated protein kinase (MAPK) signalling through β-elimination of phosphate from a key pThr with the ERK1/2 activation loop.^61^ More recently, two Dha residues, and three Dha and four Dhb residues were discovered in the matrix and capsid proteins, respectively, of human immunodeficiency virus type I (HIV-1), a contagious and deadly infectious disease.^58^ It was hypothesized that these dhAA residues are important in viral particle maturation.^58^ However, it remains to be determined whether the dhAA formation is of enzymatic or non-enzymatic origin.

dhAA residues can also be observed in some human proteins, such as the lens proteins of the eye. Due to a lack of obvious orthologues to other known phospholyases or dehydratases in the human genome, it is believed that the formation of dhAAs in human proteins is *via* non-enzymatic processes.^58^ This is the particular case for the most long-lived proteins, the lens proteins, in the human body, where Dha and Dhb formation may result from physiological conditions over time or be induced by the chemical stress caused by UV light exposure.

## 3 dhAAs in non-ribosomal peptides (NRPs)

Non-ribosomal peptides (NRPs) represent an invaluable source of diverse bioactive scaffolds with applications in agrichemicals and medicine. They are usually produced by microorganisms such as bacteria and fungi. They often have cyclic and/or branched structures and contain a great wealth of non-proteinogenic amino acids with extensive modifications, such as *N*-methyl and *N*-formyl groups or glycosylation, acylation, halogenation, and hydroxylation. On some occasions, cyclization of amino acids (*i.e.*, Ser/Thr and Cys) to the carbonyl group of the amide in the peptide backbone results in oxazoline and thiazoline motifs, respectively, which can be subjected to further oxidation or reduction.

### 3.1 NRPs containing dhAA residues

**Fig. 10:**
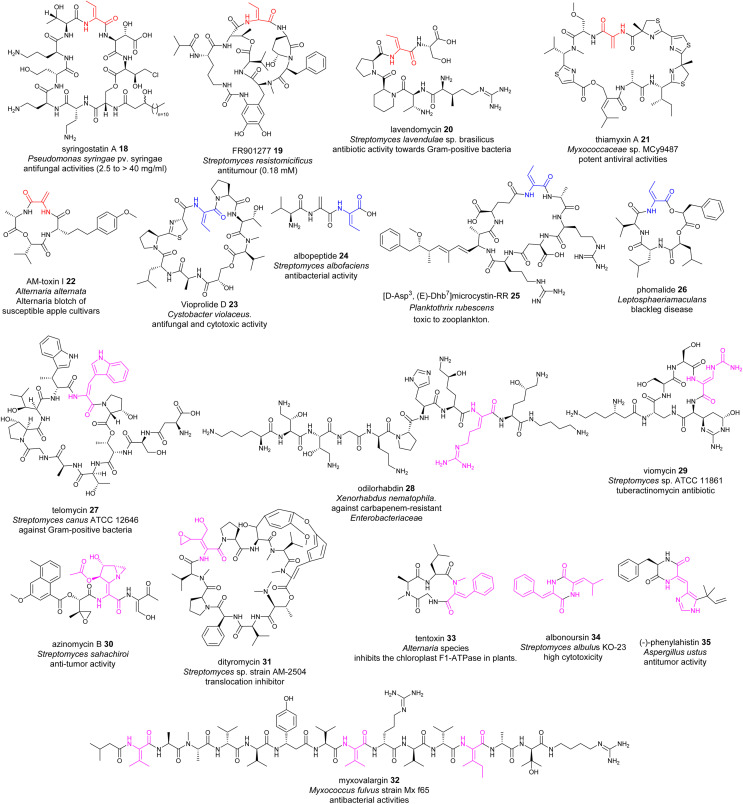
Representative structures of NRPs containing dhAA residues. dhAA residues are colour coded. Red: Dha and *Z*-Dhb; blue: *E*-Dhb; pink: other dhAA residues.

Many NRPs contain dhAA residues, with a predominant existence of Dha and *Z*-Dhb residues. This can be exemplified by the lipodepsipeptides, syringostatin A 18, isolated from Gram negative *Pseudomonas* bacteria;^62^ FR901277 19 and lavendomycin 20 from *Streptomyces* strains;^63^ thiamyxin 21 from *Myxococcaceae* strain MCy9487;^64^ microcystins discovered in various cyanobacteria;^65–67^ and the phytotoxin AM-toxin 22 isolated from the pathogenic fungal strain *Alternaria alternata*^68^ (Fig. 10). This observation may not be surprising, as such biotransformation may result from the facile/less energy-intense *anti*-elimination of protonated β-hydroxyl groups at the corresponding Ser/Thr residues *via* E1cb pathways, in a similar manner to that observed in alkene formation catalysed by dedicated dehydration (DH) domains in polyketide biosynthesis.^69^

Although less common, *E*-Dhb residues can also be found in NRPs, such as the potent antifungal and immunomodulatory agent vioprolide D 23 (ref. 15 and 70) (Fig. 10), from a myxobacterium, and more recently the tripeptide albopeptide 24, which was discovered in the culture broth of *Streptomyces albofaciens* and contains an unprecedented contiguous Dha-*E*-Dhb residue.^17^ Microcystin (MC) congeners are the most widespread class of cyanotoxins produced by cyanobacteria. 40 MC congeners have been isolated from various cyanobacteria, 10 of which contain *E*-Dhb at position 7, exemplified by [d-Asp3,*E*-Dhb7]-Microcystin-RR 25 (Fig. 10).^65,66,71,72^ The presence of *E*-Dhb residues can also be found in fungal NRPs, such as the cyclic depsipeptide phomalide 26, isolated from a fungus that causes blackleg disease (a devastating disease of several economically important brassica crops) (Fig. 10). It is worth noting that the conformations of Dhb residues in many peptidyl metabolites have not been determined. Therefore, the occurrence of *E*-Dhb residues in peptidyl natural products may be underestimated.

In some cases, the conformations of Dhb play critical roles in the biological activities of these NRPs. For example, *E*-Dhb-containing 26 is a selective phytotoxin, causing leaf spot and stem blackleg, while its synthetic isomer containing *Z*-Dhb is not a causative agent.^16^ A similar phenomenon can also be found for 24, which displays selective antimicrobial activity against vancomycin-resistant *Enterococcus faecium* hospital isolates with a MIC value of 2.98 ± 0.07 μM. Its synthetic isomer containing a *Z*-Dhb residue, however, shows no antimicrobial activity.^17^

NRPs possess other dhAA residues with great structural diversity, for example, *Z*-dehydrotryptophan (*Z*-ΔTrp) in telomycin 27 found in a *Streptomyces* strain;^73^*Z*-dehydroarginine (*Z*-ΔArg) in odilorhabdin 28 isolated from the nematode-symbiotic bacterium *Xenorhabdus nematophila*;^74,75^*Z*-β-ureidodehydroalanine in viomycin 29 (ref. 76) and aziridino[1,2-α]pyrrolidine in antitumour antibiotic azinomycins 30, produced by the culture broth of *Streptomyces* species;^77^*O*-aryl-*N*-methyl-Δtyrosine and *E*-2-amino-3-hydroxymethyl-4,5-epoxy-α,β-dehydropentanoic acid residues in dityromycin 31;^78^ ΔVal in myxovalargin A 32 from *Myxococcus fulvus* strain Mx f65;^79^ and an *N*-methyl-*Z*-dehydrophenylalanine residue (*N*-methyl-*Z*-ΔPhe) in tentoxin 33, a phytotoxic metabolite of the pathogenic fungus *Alternaria tenuis*^80^ (Fig. 10).

A range of dhAAs can also be found in cyclic diketopiperazine metabolites (DKPs). This group of metabolites display a wide range of therapeutic implications, from antimicrobial to anticancer activities.^81^ For example, both bacterial DKP albonoursin [cyclo(ΔPhe-Δ-Leu)] 34 (ref. 82) and fungal DKP dehydroHis (ΔHis)-containing phenylahistin 35 (ref. 83) display potent antitumour activity.

In many cases, these dhAA residues play important roles in the bioactivities of these NRPs as well. For example, the *Z*-ΔPhe residue is necessary for full activity of the phytotoxic tentoxin 33, which bound to chloroplast F1-ATPase during structural studies of the tentoxin-inhibited CF1-complex.^80^ GE82832/dityromycin 31 blocks the EF-G-catalysed movement of peptidyl-tRNA and mRNA from the ribosomal A-site to the P-site, without preventing the ribosomal binding of the elongation factor. Crystal structures of the antibiotics in complex with the bacterial 70S ribosome demonstrated that these antibiotics bind to the shoulder of the bacterial 30S subunit and interact exclusively with bacterial ribosomal protein S12 on the small subunit, thereby inhibiting EF-G-catalysed translocation by disrupting a critical contact between EF-G and S12 that is required to stabilize the post-translocational conformation of EF-G.^78^ It was found that the antibiotics form a contact with the His76 of S12, forming a hydrogen bond between the delta nitrogen of His76 and the hydroxyl group of the *E*-2-amino-3-hydroxymethyl-4,5-epoxy-α,β-dehydropentanoic acid residue of GE82832/dityromycin 31.

### 3.2 Transient dhAAs in NRP-originating metabolites

**Fig. 11:**
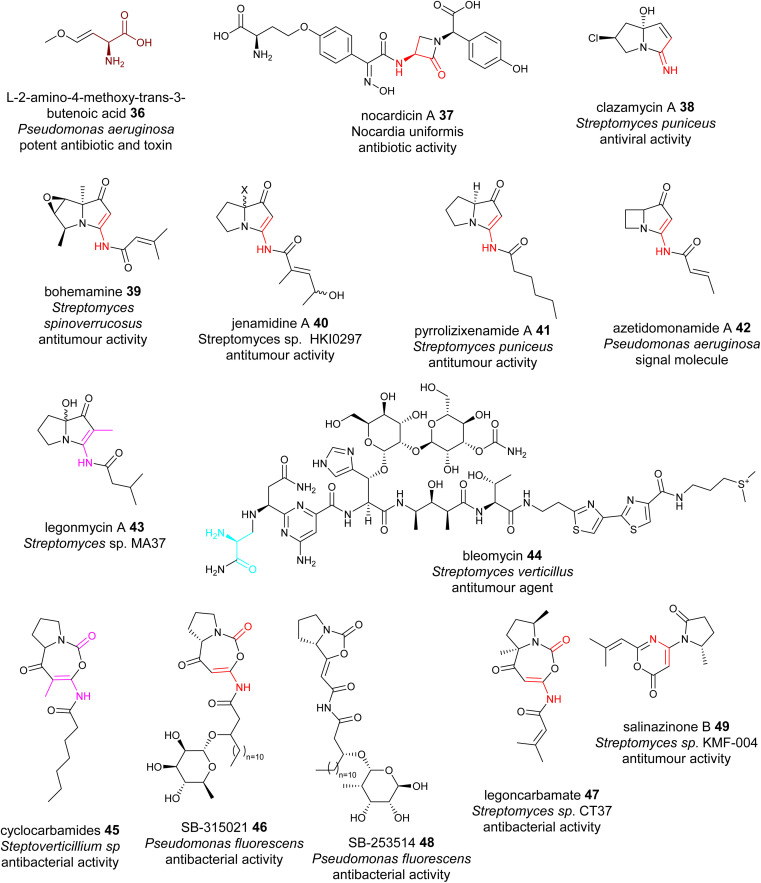
Representative structures of NRPs containing transient dhAA residues. Transient dhAA residues are colour coded. Transit Dha: cyan;Dha: red; Dhb: pink; other dhAA residues: brown.

dhAAs can be further modified during NRP maturations, a similar process to the extensive PTMs in RiPPs. Oxyvinylglycines are nonproteinogenic amino acids with a core β,γ-vinyl ether and a variety of alkoxy substituents.^84^ They are potent inhibitors of the eliminase subgroup of pyridoxal phosphate (PLP)-dependent enzymes. For example, 2′-aminoethoxyvinylglycine inhibits 1-aminocyclopropane-1-carboxylate synthase, a key PLP-dependent enzyme in the biosynthesis of the plant growth hormone, ethylene. It has been developed as the key ingredient of the commercial plant growth regulator, ReTain (Valent). During the biosynthetic study of l-2-amino-4-methoxy-*trans*-3-butenoic acid (AMB) 36, an oxyvinylglycine (Fig. 11), a putative intermediate containing a α,β-ΔGlu derivative was proposed to be formed.^84^

Chemo-enzymatic studies demonstrated that the monocyclic β-lactam motif of nocardicin A 37 (Fig. 11) is derived from an addition between the amine group of l-(*p*-hydroxyphenyl)glycine (l-pHPG) and a transient Dha of the growing peptidyl intermediate.^85–87^

Pyrrolizidines are a group of heterocyclic compounds consisting of two-fused 5-membered rings with a nitrogen atom at the bridgehead.^88^ Naturally occurring pyrrolizidine alkaloids (PAs) are mainly produced by plants as a defence mechanism against insect herbivores. More than 660 PAs and derivatives have been found in over 6000 plants over the world and 3% of the world's flowering plants contain PAs. Half of PAs, mainly the unsaturated PAs, are hepatotoxic and carcinogenic.^88^ Compared to a large number of plant PAs, only a handful PAs have been isolated from bacteria. To date, approximately 30 bacterial PAs have been discovered, including clazamycins 38, bohemamine 39, jenamidine A 40, pyrrolizixenamide 41 and azetidomonamide 42 from various bacterial strains (Fig. 11). Many bacterial PAs display potent anticancer and antimicrobial activities.^88^ It was demonstrated that a transient Dha (Dhb in the case of legonmycins) residue is formed during the biosynthesis.^89–95^

Transient dhAA residues could be involved in the formation of pyrimidine motifs during the biosynthesis of the anticancer drug bleomycin 44. Pyrimidines are aromatic six-membered heterocycles containing two N atoms in the ring, and are commonly found in medically relevant compounds. Due to the importance in anticancer therapeutics, 44 (Fig. 11) has been extensively investigated since 2000.^96^ Through comparative analysis of the BGCs and pathways of bleomycin and its analogous NPs, it was hypothesized that a transient Dha derived from Ser could be formed, followed by addition of the amine group of the activated l-Asn, resulting in a covalent bridging, a similar process to the first step of forming a monocyclic β-lactam motif. However, how this Dha-involving reaction could lead to the formation of pyrimidine motifs has remained enigmatic.^97^

[5+5] and [5+7] cyclocarbamate NPs provide the inspiration for the first-in-class synthetic phospholipase inhibitor darapladib. Only five have been reported, including natural lipocyclocarbamate 45 isolated from an unidentified *Streptoverticillium* sp.,^98^ SB-315021 46 and SB-253514 47 from various *Pseudomonas* strains, and more recently legoncarbamate 48 from an environmental isolate of *Streptomyces* sp CT37 (Fig. 11).^99^ Interestingly, many of these compounds with [5+7] ring systems were co-metabolites with either bacterial PAs or 48 with a [5+5] ring system (Fig. 11).^100^ It is likely that formation of these [5+7] cyclocarbamates is similar to bacterial PAs, involving a transient Dha to form a key [5+6] indolizidine intermediate. All isolated carbamates with either [5+7] or [5+5] systems display potent antibacterial activities.

The final example of NPs containing a transient Dha is salinazinone A 49 (Fig. 11), which contains an unusual pyrrolidinyl-oxazinone isolated from solar-saltern-derived *Streptomyces* sp. KMF-004.^101^ The structures of 49 two compounds contain 2-methylpropenyl-1,3-oxazin-6-one bearing 1-oxopyrrolidinyl substituents. It was postulated that 49 are derived from the oxidative re-arrangement of PAs.

### 3.3 Enzymes responsible for the formation of dhAA residues

NRPs are synthesized by large modular multienzyme complexes, NRPSs, consisting of repeating domains grouped into modules that cooperate to assemble and modify diverse peptides. NRPSs have three core catalytic domains: the adenylation (A) domain, responsible for the selection and activation of each amino acid; the thiolation (T) domain, which captures the activated amino acids using its phosphopantetheine (ppant) ‘arm’; and the condensation (C) domain responsible for linking one amino acid residue (acceptor) with the growing peptidyl intermediate (donor). Release of the fully assembled peptide chain typically involves a thioesterase domain that catalyses either hydrolysis or macrocyclisation to produce a linear or cyclic peptide, respectively. C domains commonly possess a V-shaped pseudodimer consisting of N- and C-terminal lobes and a highly conserved motif H**H**XXXDG located in the junction of the lobes.^102^ Apart from classical condensation reactions, C domains also play highly diverse roles during NRP biosynthesis, such as epimerization (E), cyclization (Cy), lipidation (Starter), dual C/E and hydrolysis.^103^

C domains in NRPSs can be grouped into six main clades, ^start^C, ^L^C_L_, ^D^C_L_, C/E, E and Cyc, based on substrate specificity.^104,105^ Interestingly, there is a small clade called C_modAA_, which includes only two experimentally uncharacterized C domains identified in the BGC of microcystin and bleomycin. It was postulated to be involved in the incorporation of dhAAs.^105^

**Fig. 12:**
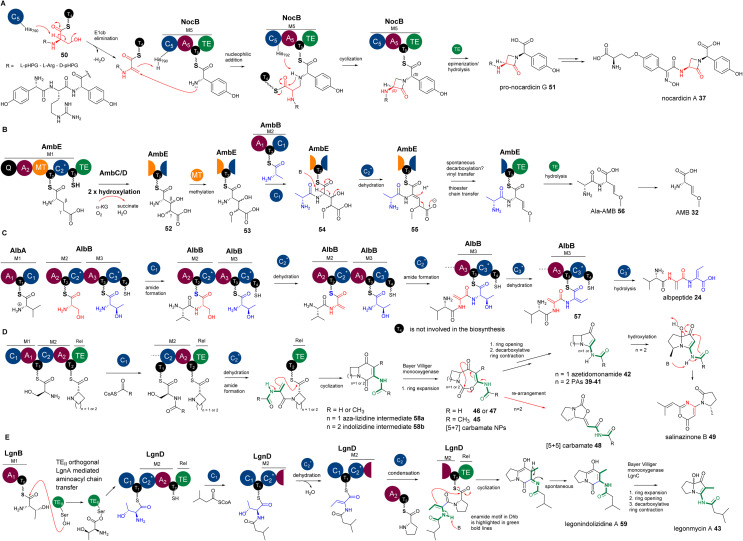
(A) The formation of β-lactam in nocardicins resulting from the addition reaction between the amine of l-pHPG and the transient Dha residue in the growing peptidyl intermediate. (B) The β,γ-alkene motif in AMB, resulting from the decarboxylation of the proposed intermediate containing an α,β-ΔGlu derivative, followed by a vinyl shift. (C) The Dha and *E*-Dhb formations in the biosynthesis of albopeptide, catalysed by AlbB-C_2_ and AlbB-C_3_ domains, respectively. (D) The proposed biosynthetic pathway of bacterial PAs and azedomonamide. The final Te domains of the colinear corresponding NRPSs are proposed to activate the nucleophilicity of the enamide motif (highlighted with green bold lines) in the corresponding Dha for the final cyclization to yield a [5+6] indolizidine ([4+6] aza-lizidine in the case of azedomonamide) intermediate, followed by multistep transformations of a Baeyer–Villiger reaction involving ring expansion, hydrolytic ring opening and decarboxylation-driven ring contraction to finally provide a [5+5] ([4+5] in the case of azedomonamide) ring system. During the ring expansion, [5+7] carbamate intermediates could be trapped and released to provide 45–47. Further re-arrangement of the [5+7] ring systems will give [5+5] carbamate 48. In a divergent pathway, an unidentified oxidative enzyme could re-arrange the [5+5] PA systems into pyrrolidinyl-oxazinone, 49. (E) The proposed biosynthetic pathway of legonindolizidine A 59, the intermediate of legonmycin A 43. The presence of LgnA is necessary to transfer LgnB-T_0_-tethered l-Thr to LgnD-T_1_ for LgnD-C_2_-catalysed dehydration/condensation.

The first biochemically characterized DeHydrating C domain (DHC) was the one in the 5th module (M5C) of NocB in the pathway of nocardicins.^85^ Two multidomain NRPSs, NocA and NocB, are responsible for assembly of a pentapeptide, l-pHPG-l-Arg-d-pHPG-l-β-lactam-d-pHPG, followed by post-modification to remove the l-pHPG-l-Arg dipeptide at the late stage of the biosynthesis of nocardicins.^85^ Incubation of *holo*-M5, ATP and l-pHPG with NocB-T_4_-tethered 50 resulted in the production of β-lactam-containing pro-nocardicin G 51 (Fig. 12A), the precursor of nocardicins. When T_4_-tethered l-pHPG-l-Arg-d-l-pHPG-Dha was incubated with *holo*-M5, ATP and l-pHPG, the β-lactam motif was also formed. Taken together, these analyses strongly indicated that the conversion of the Ser residue of 50 into Dha, catalysed by the C_5_ domain of M5 (M5C), is key to the formation of the β-lactam motif (Fig. 12A).^85^ The active site of M5C contains a rather unique motif of **H**^790^H**H**^792^xxxDG. Changing H^790^ and H^792^ to Ala by site-directed mutagenesis (SDM) resulted in complete abolishment of pro-nocardicin G production, indicating the key roles of both His residues during the β-lactam formation.^85^ An E1cb pathway was proposed for the dehydration of Ser to transient Dha catalysed by M5C, as evidenced in *in vitro* reconstitution coupled with isotopic labelling experiments.^87^ Further evolutionary analysis^106^ demonstrated that M5C is a rather special member of the ^D^C_L_ domains that evolved to possess a dehydrating function. It was found that it retains its ancestral function as a competent ^D^C_L_ catalyst to form peptidyl bonds only when d-Ser-containing tetrapeptidyl donors are provided.^106^ Interestingly, the upstream T_4_ domain is denoted as T_E_, a donor T domain for an E and ^D^C_L_ domain. It has the consensus motif of GGD̲SI. The donor T domain (T_c_) for a ^L^C_L_ domain normally has the motif of GGH̲SL.^107^

Other characterized DHCs, however, were found to phylogenetically belong to C_modAA_ domains and possess a classical motif of H**H**xxDG in their active sites. *In vitro* pathway reconstitution of AMB 36 (ref. 84) demonstrated that four enzymes are required to produce this rather simple molecule, including two FeII/aKG dependent oxygenases, AmbC and AmbD, and two multidomain NRPSs, AmbB and AmbE, with the arrangements of A-T-C and Q-A-MT-T_1_-C_2_-T_2_-TE (where Q has unknown function and MT is a methyltransferase domain), respectively. AmbB-A and AmbE-A activate l-Ala and l-Glu, respectively (Fig. 12B). Using deuterium-labelled amino acid precursors and the chemical capture method using excess cysteamine,^108^ the biosynthetic pathway was deduced, where two hydroxylases, AmbC and AmbD, separately install two hydroxyl groups in AmbE-T_1_-bound Glu to provide 3,4-dihydroxy-Glu intermediate 52. AmbE-MT catalyses *O*-methylation on the 4-hydroxyl group of 53, followed by condensation with AmbB-tethered Ala to form the dipeptide Ala-l-3-hydroxyl-4-methoxy-Glu thioester 54. The AmbE-C_2_ domain catalyses 2,3-dehydration on 55 to provide a dipeptide containing a transient dhAA. This unstable intermediate is subjected to a decarboxylation-driven vinyl shift and subsequent hydrolysis to provide the pro-drug, Ala-AMB 52 (Fig. 12B). It was proposed that 52 is a self-protective molecule, marking the α-amino group of AMB for the producing strain, which could be removed during the export of Ala-AMB.^84^

Inspection of the structure of albopeptide 24 suggested that l-Ser and Thr could be the precursors of the Dha and *E*-Dhb residues, respectively.^17^*E*-Dhb residues in peptidyl metabolites have long been postulated to originate from the facile *anti*-elimination of l-*allo*-Thr, of which the α-proton and the β-hydroxyl group are in the opposite configurations to allow the *anti*-elimination to occur in a concerted manner.^15^ Biochemical analysis demonstrated that two multidomain NRPSs, AlbA and AlbB, are required for the production of 24.^17^ The substrates of AlbA and AlbB are l-Val, l-Ser and l-Thr instead of l-*allo*-Thr. Although only two peptide bonds are present in 24, three condensation domains were observed, suggesting that some of these C domains may be involved in unconventional processes. Indeed, further phylogenetic analysis of these C domains revealed that, while C_1_ was predicted to be a canonical ^L^C_L_ domain, both C_2_ and C_3_ are DHC domains. Incubation of *holo*-AlbA and AlbB with l-Val, l-Ser, and l-Thr resulted in the production of 24. In the presence of excess cysteamine,^108^ MS profiling of various *in vitro* assays confirmed that, while AlbB-C_2_ catalyses the dehydration of Ser to Dha and the condensation between the resulting Val-Dha dipeptidyl thioester and the downstream Thr residue, AlbB-C_3_ catalyses the unique dehydration of l-Thr to *E*-Dhb and the hydrolysis on the final intermediate of Val-Dha-*E*-Dhb thioester 57. It was demonstrated that the dehydration of Ser/Thr must occur prior to the condensation of the resulting dehydropeptidyl donor intermediate and the downstream amino acid acceptor, or hydrolysis (Fig. 12C). Such timing was also observed in the biosynthetic study of AMB, where the dehydration occurs at AmbE-T_1_ catalysed by the DHC, AmbE-C_2_, to generate the final intermediate, followed by transthioesterification of the resulting product from AmbE-T_1_ to the downstream AmbE-T_2_.^84^ This allows the final hydrolysis by AmbE-TE to provide Ala-AMB^84^ (Fig. 12B).

Intriguingly, a similar phenomenon in the timing of condensation was also observed for C/E domains. Biosynthetic studies of the biosurfactant arthrofactin demonstrated that the activated l-amino acid in the growing peptidyl chain should be epimerized to the D-counterpart first, followed by condensation with the downstream amino acid residue.^109^ How these C/E and DHC domains control the timing of epimerization/dehydration and condensation, respectively, remains to be determined.

It is worth noting that AlbB-C_3_ is the first that was biochemically confirmed to act on l-Thr to provide *E*-Dhb. Unlike the *anti*-elimination in organic synthesis, where the α-proton and β-hydroxyl are on the opposite faces, production of *E*-Dhb from l-Thr requires the α-proton and β-hydroxyl group of l-Thr to be on the same face for *syn*-elimination, as shown in Fig. 2D. This raises the question of how AlbB-C_3_ handles l-Thr. Further co-crystallography studies with the substrates are required.

Enamides are considered versatile synthetic building blocks in organic synthesis, particularly for carbon–carbon bond formation.^18^ Compared to enamines, which are highly sensitive toward hydrolysis, enamides are shelf-stable enamine surrogates that display a diminished enaminic reactivity, but an increased stability, being masked by the electron-withdrawing carbonyl group. The delocalization of the lone-pair electrons of the nitrogen atom into the adjacent alkene confers a certain degree of nucleophilic properties. Consequently, enamides have been successfully utilized as reactive nucleophiles in enantioselective reactions.^110^

However, to the best of our knowledge, the utility of enamide nucleophilic functionality in dhAA residues has not been explored in organic synthesis. Interestingly, biochemistry driven by enamide functionality for dhAA residues is also hardly found in nature. The only examples of dhAA enamide nucleophilic substitution are in the biosynthesis of bacterial PAs. It has been demonstrated that bacterial PAs originate from multidomain NRPSs, first generating bicyclic indolizidine intermediate 58, followed by the multistep biotransformation of ring expansion, ring opening and ring contraction, catalysed by single FAD-dependent monooxygenases, to finally provide pyrrolizidine frameworks (Fig. 12D).^89–95^ During the formation of indolizidine intermediates, a transient dhAA residue is generated, followed by a dhAA-enamide-driven C–C formation, cyclized by the last type I thioesterase (TEI) domain (Fig. 12D).^89–95^

While the biosynthetic pathways of other bacterial PAs have canonical NRPS complexes and follow the collinearity rules, the assembly of legonmycins is rather unusual. It includes two NRPS proteins, LgnB and LgnD, with domain arrangements of A_1_-T_0_ and C_1_-T_1_-C_2_-A_2_-T_2_-TE, respectively (Fig. 12E). Another unique feature is that the biosynthesis requires the presence of LgnA, a type II thioesterase (TEII) orthologue, to catalyse aminoacyl chain transfer between T_0_ and T_1_ domains on two separate NRPS subunits.^112^ It was demonstrated that the DHC, LgnD-C_2_, catalyses the dehydration of l-Thr at the LgnD-T_1_ domain (Fig. 12E). l-Thr, first activated by LgnB-A_1_ and loaded in LgnB-T_0_, must be transferred by LgnA to the LgnD-T_1_ domain for the dehydration (Fig. 12E). The reason why LgnD-C_2_ only recognises LgnD-T_1_-tethered IV-Thr (but not LgnB-T_0_) for dehydration may lie in the sequence difference between LgnB-T_0_ and LgnD-T_1_. LgnB-T_0_ is a typical T_c_ domain with a motif of GGH̲SL, which is supposed to accommodate the condensation between l-Thr and acyl-CoA, catalysed by the ^start^C domain, LgnD-C_1_. Such a design is, however, not suitable for the downstream dehydration catalysed by the DHC domain, LgnD-C_2_. Therefore, it is a necessity to insert an extra T_E_ domain, LgnD-T_1_, with a signature of GGD̲Sx (x = I or V), that is specialised for LgnD-C_2_-mediated dehydration. To shuttle the aminoacyl chain between two types of T domains, the recruitment of LgnA to the *lgn* biosynthetic cluster is required to enable the evolution of this NRPS pathway. LgnA also maintains its hydrolytic function to remove the aberrant LgnB-T_0_-tethered IV-Thr intermediate resulting from non-selective condensation between IV-CoA and a l-Thr unit, which is catalysed by the promiscuous C_start_ domain, LgnD-C_1_. Intriguingly, when LgnA is absent in *in vitro* reconstitution experiments (no aminoacyl chain transfer occurs), LgnD-C_2_ still maintains its competent ^L^C_L_ function (but not dehydration) to catalyse a condensation between this misprogrammed LgnB-T_0_-tethered IV-Thr and LgnD-T_2_-tethered L-Pro. This generates a shunt intermediate, NRPS-tethered IV-Thr-Pro, which cannot be recognised by LgnD-TE for final cyclization or hydrolysis, thus blocking the assembly line (Fig. 12E).^112^

The backbones of cyclocarbamates are derived from indolizidine intermediates.^100,111^ Thus, the involvement of a transient Dha should be the same as what occurs in bacterial PAs. Subsequent insertion of an oxygen atom *via* Baeyer–Villiger ring expansion pathways could give [5+7] systems 45–47 (Fig. 12D). This [5+7] ring system could undergo further ring re-arrangement to give the [5+5] bicyclic carbamate 48 with a different geometry of ring closure (Fig. 12D).^100,111^ An unidentified oxidative enzyme could further re-arrange the [5+5] PA system into a pyrrolidinyl-oxazinone to generate 49 (Fig. 12D).^101^

**Fig. 13:**
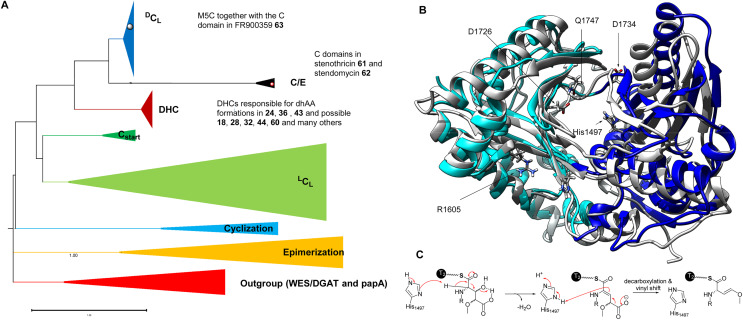
(A) Rooted phylogeny of the C domain superfamily using bacterial wax ester synthase (WES) and polyketide-associated protein A5 (papA) enzymes as outgroups to root the phylogeny, as these two superfamilies show high structural and functional similarity to C domains. The DHC domains, such as AmbE-C_2_, AlbB-C_2_, AlbB-C_3_, LgnD-C_2_, and many others, belong to the DHC clade while M5C belongs to the ^D^C_L_ clade. Interestingly, C domains possibly responsible for dhAA formations in both 61 and 62 are likely in the C/E clade, suggesting the presence of a new variation of DHCs. (B) The overlapped structures of an AmbE-C_2_ domain and a characterized C domain involved in the biosynthesis of fuscachelin. The N-lobe and C-lobe of the AmbE-C_2_ domain are colour coded in light blue and blue, respectively. The amino acid residues with charged side chains, which were demonstrated to be involved in the reaction, are highlighted as sticks. (C) A proposed mechanism of dehydration of a Glu derivative, followed by the decarboxylation and vinyl shift.

Rooted phylogenetic analysis has indicated that, while M5C is a special member of the ^D^C_L_ domains, other characterized DHCs, such as AmbE-C_1_, LgnD-C_2_, AlbB-C_2_ and AlbB-C_3_, are clustered (Fig. 13A). Two clades are distinguishable, relating to early divergence in C-domain functions. While ^L^C_L_ and C_start_ domains can be grouped together, DHC, C/E and ^D^C_L_ domains form a separate group.^106^ Interestingly, the members of DHC and C/E catalyse the loss of C-α stereochemistry as the first chemical step and display configuration control over the timing of condensation with the downstream l-donors.^106^ This could be further supported by the observation of sequence divergence of the upstream T donors, which can be separated into T_C_ domains for ^L^C_L_ and C_start_, and T_E_ domains for E, DHC, C/E and ^D^C_L_, that are either co-evolved with ^L^C_L_ and C_start_ or inherited from the association with E-domains, respectively.

Very recently, the first structure of the DHC AmbE-C_2_, from AMB biosynthesis, was reported.^113^ The overall structure of AmbE-C_2_ displays high similarity to other characterized C domains, as a V-shaped pseudodimer consisting of N- and C-terminal lobes (Fig. 13B). The junction of the lobes forms the classic active-site tunnel that connects the donor and acceptor T-domain-binding sites with the active site, containing the characteristic motif of H**H**xxxDG (H1496–G1502). Structural modelling with the substrate, AmbE-T_1_-tethered pre-Ala-AMB, allowed identification of several charged residues, R1605, D1726, D1728, D1734 and E1736, in the vicinity of the active site of AmbE-C_2_, which potentially protonate the β-hydroxyl group of the 3-hydroxy-4-methoxy-Glu residue in the substrate (Fig. 13C). Alanine scanning mutagenesis on AmbE-C_2_ demonstrated that changing H1497, R1605, D1726, D1734 and Q1747 to Ala results in significant loss or complete abolishment of the expected products. However, these residues are not conserved in other DHCs. This may not be surprising, as the substrate of AmbE-C_2_ contains a rather unusual amino acid residue. Many DHCs catalyse the conversion of canonical amino acids (*i.e.*, l-Ser/Thr) to the corresponding dhAAs (*i.e.*, Dha/Dhb, respectively).

### 3.4 dhAA formation from putative enzymatic activities in NRP pathways

**Fig. 14:**
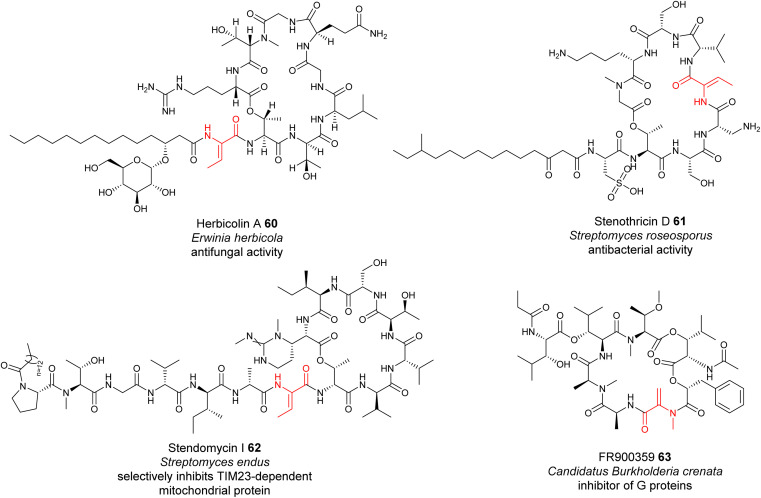
Representative structures of NRPs containing dhAA residues, the biochemical origin of which remain to be determined.

The formation of many dhAAs in NRPs may occur *via* similar transformations to those of DHCs for 24, 36 and 43. Such examples may be found in the biosynthetic pathways of cyclic lipopeptides, such as the antifungal herbicolin A 60 (ref. 114) (Fig. 14), and linear peptides such as odilorhabdin 28 (ref. 74) and myxovalargin 32 (ref. 79) (Fig. 10). Phylogenetic analysis suggested that the C domains responsible for linking the dhAA residues (*Z*-Dhb in 60, ΔArg in 28, and ΔVal-2, ΔVal-9 and *E*-ΔIle-13 in 32) with the downstream amino acid residues belong to the cluster of DHCs (Fig. 13A). Interestingly, the upstream T domains of these putative DHCs also contain the characteristic motif of the T_E_ subgroup, in line with the observations for 24, 36 and 43. In the case of dhAAs in 28 and 32, it is likely that the putative hydroxylases are responsible for the in-line installation of a hydroxyl group on the corresponding proteinogenic amino acid residues in the growing peptidyl chain, to give the transient β-hydroxyl products first, followed by dehydration and subsequent condensation.^75^

However, this may not be the case for the dhAA residues in the antibiotic stenothricin 61 (ref. 115) and the antifungal agent stendomycin I 62 (ref. 116) (Fig. 14). 61 is a linear octapeptide structure containing a Dhb-5 residue of unknown stereochemistry and an unusual cysteic acid residue (Fig. 14). 62 contains a heptadepsipeptide ring and a heptapeptide linear chain with a Dhb-7 residue (Fig. 14). Analysis of the corresponding NRPS assembly line suggested that the C domains possibly responsible for the connection between Dhb in the growing peptidyl intermediate and the downstream amino acid residues belong to the C/E clade (C_6_ domain for 61 (Genbank ID EFE73312.1) and C_8_ domain for 62 (Genbank ID EFL21631.1)), suggesting the existence of special dehydrating C/E domains.

A similar case can be also found in the biosynthesis of the cyclodepsipeptide FR900359 63, which was originally isolated from the evergreen plant *Ardisia crenata sims* (*Myrsinaceae*).^117,118^63 is a potent inhibitor of the Gq subfamily of guanine nucleotide-binding proteins (G proteins), which can be used to treat complex diseases such as asthma, inflammation and cancer.^119^ A recent study indicated that the molecule is actually produced by the unculturable bacterial endophyte *Candidatus Burkholderia crenata*, located in the leaf nodules.^120^ One of the C domains, although being a member of ^D^C_L_ domains, was predicted to link the *N*-methyl-Dha-containing growing peptidyl chain with the downstream l-Ala.^121^ The formation of this Dha in FR900359 would require further investigation.

Other oxidative enzymes may also be responsible for dhAA formation in different NPs. This can be exemplified by various dhAA residues in DKPs. It has been well-established that most bacterial DKPs originate from non-ribosomal tRNA-dependent cyclodipeptide synthase pathways.^122^ Some of these DKPs, such as albonoursin 34, contain dhAA moieties. In these pathways, tRNA-dependent cyclodipeptide synthases catalyse the synthesis of saturated DKP, followed by cyclic dipeptide oxidases to introduce dhAA residues.^122^ Fungal DKPs, however, are derived from NRPS assembly lines. Gene inactivation and feeding experiments indicated that the putative cytochrome P450 enzyme EchP450 is essential for the formation of ΔTrp in the echinulin family alkaloids.^123^ However, the enzyme responsible for the Dha residue remains elusive. Another case can be found in the biosynthesis of viomycin 29, which is the first member of the tuberactinomycin family and is used in a drug cocktail for the treatment of multidrug-resistant tuberculosis. It was proposed that the FAD-dependent dehydrogenase VioJ could catalyse the dehydrogenation of the protein-tethered *N*-acyl-2,3-diaminopropionyl unit in the growing peptidyl chain to provide β-amino-ΔAla, followed by carbamoylation catalysed by the putative carbamoyltransferase VioL to finally furnish a *Z*-β-ureido-ΔAla motif.^76,124^

There are many other dhAA residues in bacterial NRPs, the formation of which has remained unanswered. This can be exemplified by the *Z*-ΔTrp in telomycin 27, aziridino[1,2-α]pyrrolidine in azinomycins 30, and *O*-aryl-*N*-methyl-ΔTyr and *E*-2-amino-3-hydroxymethyl-4,5-epoxy-α,β-dehydropentanoic acid residues in dityromycin/GE82832 31 (Fig. 10).

## 4 dhAA residues from unidentified biosynthetic pathways

**Fig. 15:**
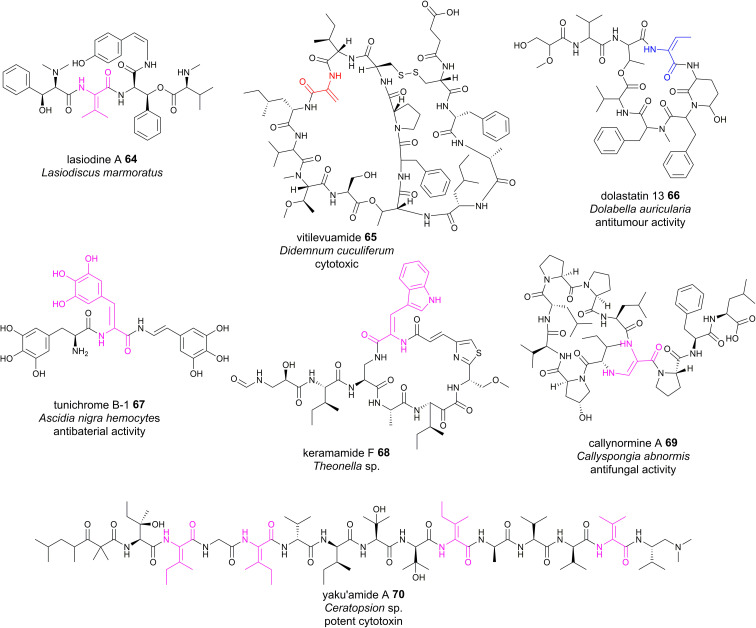
Representative structures of NRPs containing dhAA residues, the biosynthesis of which remains to be determined.

Although plants are prolific producers of bioactive cyclopeptides, dhAA-containing peptides are exceedingly rare. Lasiodine A 64 (Fig. 15) is a linear tetrapeptide isolated from the leaves of *Lasiodiscus marmoratus*, a small plant genus in the family *Ramnaceae*^125^ This was the first report of a ΔVal residue in a natural compound. The bio-origin of this dhAA-containing peptidyl metabolite is unknown. Given that plants lack genes encoding multidomain NRPSs, it is possible that lasiodine A may be derived from a ribosomal origin. It is known that many plant cyclic RiPPs contain unusual C–N, C–O or C–C connections at amino acid side chains. Recent studies demonstrated that these RiPPs originate from a growing member of RiPPs, of which the precursor peptides carry multiple core motifs for various peptides.^126^ In addition to these CPs, the precursor peptides have an N-terminal signal sequence and a C-terminal BURP domain (BURP is an acronym for the four initially identified examples of the domain: BNM2, USP, RD22, and PG1b). In these systems, the core peptides are fused to the copper-dependent autocatalytic BURP domains.^127–130^ These BURP domains are oxidative cyclases that install the characteristic amino acid side-chain cross-links. In this regard, dhAA residues in plant RiPPs may be derived from the action of BURP domains.

Marine animals are a treasure trove of bioactive dhAA-containing peptides. For example, vitilevuamide 65 (Fig. 15), a bicyclic Dha-containing tridecadepsipeptide, was isolated from two marine ascidiands, *Didemnum cuculiferum* and *Polysyncranton lithostrotum*. 65 possesses anticancer activity and is cytotoxic towards several human tumour cell lines.^131,132^*Z*-Dhb-containing dolastatin 13 66 (Fig. 15)^132^ was originally isolated from the sea hare *Dolabella auricularia*, and possesses potent Ser protease inhibitory properties. Apart from common Dha and Dhb residues, peptides from these sources are also enriched with other dhAAs. For example, dehydroPhe (ΔPhe) residues with various hydroxylation patterns were found in tunichromes 67 isolated from tunicates and celenamides from the sponge *Cliona celata*. Keramamide F 68 (Fig. 15) is a cyclic *Z*-ΔTrp-containing heptapeptide isolated from the Okinawa marine sponge *Theonella* sp. 68 shows cytotoxicity against human epidermoid carcinoma KB cells and murine lymphoma L1210 cells. A very unusual dhAA, *Z*-2,3-diaminoacrylic acid, was found to be present in callynormine A 69 (ref. 133) isolated from the sponge *Callyspongia abnormis* and *C. aerizusa* (Fig. 15). This residue plays a critical role in providing the linkage to the peptide side chain. The linear peptide yaku'amide A 70 (Fig. 15) was isolated from the deep-sea sponge *Ceratopsion* sp. It contains several unusual β-*tert*-hydroxy amino acids and two *Z*-ΔIle residues, one *E*-ΔIle residue and one ΔVal residue.^134^ It strongly inhibits the growth of P388 murine leukemia cells (IC_50_ = 14 ng mL^−1^).

Although many of these dhAA-containing peptides were originally isolated from marine animals, they are most probably of bacterial/cyanobacterial/fungal origins, as these invertebrates have acquired very powerful chemical defences by careful selection and/or biosynthetic manipulation of their hosted bacterial/cyanobacterial/fungal symbionts.

## 5 Outlook and future perspectives

Many dhAA-containing peptidyl natural products have an enormous yet unexploited potential for lead-compound discovery.^135^ In many cases, dhAA moieties and their conformations in these compounds are essential for their bioactivities. However, the great bottleneck to develop these bioactive compounds is producing them in large quantities for preclinical and clinical development. Moreover, the structural complexity of these compounds makes them difficult to obtain *via* chemical synthesis, preventing useful structural activity relationship (SAR) studies to determine key bioactive moieties. Nevertheless, several dhAA-containing metabolites that possess potent *in vitro* activity have been chemically modified for lead optimisation to increase *in vivo* efficacy and further enhance pharmacokinetic properties without diminishing activity. For example, dhAA-containing odilorhabdin 28 and phenylahistin 35 are the inspiration for the clinical candidate NOSO-503 for carbapenem-resistant *Enterobacteriaceae* and the antitumour lead-compound plinabulin, respectively.^136^

dhAA moieties in peptidyl molecules display versatile chemistries due to their unique push–pull electronic features. Such architectures in peptides allow access to unnatural amino acid derivatives and complex ring systems through late-stage modifications, offering robust chemical handles for structure–activity relationship studies. For example, the application of photocatalysis under visible light radiation to functionalise dhAA derivatives has become a new trend in biorthogonal strategies, as it often offers a versatile and controllable modification tool.^3^ In turn, such visible-light-driven bioconjugations with fluorescence tags under mild conditions in aqueous solutions may assist the discovery of previously unnoticed dhAA-containing natural products during microbial fermentation. Investigations of novel dhAA-containing natural product biosyntheses would allow identification of new dhAA-modifying enzymes that potentially display chemical transformations not previously observed in natural product biosynthesis.

## 6 Conflicts of interest

The authors declare no conflicts of interest.

## Supplementary Material
